# Longitudinal measurement invariance of the Working Alliance Inventory - Short form across coaching sessions

**DOI:** 10.1186/s40359-022-00968-5

**Published:** 2022-11-23

**Authors:** Marjolein Stefens, Eefje Rondeel, Jonathan Templin, David Brode, Eddy de Waart, Rendel de Jong, Jacobien ten Hoeve-Rozema, Alexander Waringa, Jennifer Reijnders, Nele Jacobs, Johan Lataster

**Affiliations:** 1 Department of Lifespan Psychology, Faculty of Psychology, Open Universiteit, P.O. Box 2960, 6401 Heerlen, DL The Netherlands; 2 Freelance Professional, Schimmert, The Netherlands; 3 Department of Psychological and Quantitative Foundations, University of Iowa, Iowa City, USA; 4 Scientific Research Committee, European Mentoring & Coaching Council The Netherlands (NOBCO), Nijkerk, The Netherlands; 5 Research and Development Department, UNLOQ Academy, Tilburg, The Netherlands; 6 Department of Psychiatry and Psychology, School for Mental Health and Neuroscience, Maastricht University Medical Centre, Maastricht, The Netherlands

**Keywords:** Coaching, Working alliance inventory, Longitudinal measurement invariance, Psychometrics

## Abstract

**Background:**

Throughout the psychotherapeutic and coaching literature, the client-therapist or coach-coachee working alliance has been highlighted as key force driving positive outcome. The Working Alliance Inventory Short form (WAI-S) for coaching charts the quality of working alliance throughout coaching sessions and is broadly applied in coaching research. Due to a shortfall in research on psychometric properties of the WAI-S, the purpose of this study was to examine (a) if the theorized three-factor structure of the 12-item WAI-S forms a solid representation of the dimensions of working alliance in coaching, and (b) longitudinal measurement invariance (LMI) of the WAI-S.

**Method:**

Data were collected in a two-wave study design comprising a main study sample of *N* = 690 Dutch coachees that completed the questionnaire at the first measurement, of which *N* = 490 also completed the questionnaire at the second measurement. Post hoc sensitivity analysis was performed based on the original sample, lacking additional information on covariates, and included both completers and dropouts, comprising *N* = 1986 respondents at T1, and *N* = 1020 respondents at T2.

**Results:**

Confirmatory factor analyses evidenced best fit of the three-factor model in comparison to one-, and two-factor models at both time points. Despite the fact that multigroup confirmatory factor analysis detected non-invariant intercepts, our findings overall supported measurement invariance across coaching sessions.

**Conclusions:**

As decisions in both clinical and scientific practices generally rely on outcome assessment of interpersonal change in scores on the same measure over time, we believe our findings to be of contributing value to the consolidation of interpretation and accuracy of scorings on the WAI-S in coaching.

**Supplementary Information:**

The online version contains supplementary material available at 10.1186/s40359-022-00968-5.

## Introduction

Decades of research on the active ingredients of therapeutic interventions have converged on the identification of the professional working alliance between client and therapist as a leading common factor [1, 2], asserting that a stronger alliance relates to greater therapeutic change [3]. As coaching and psychotherapy are both based on helping relationships and can be categorized as personal interventions [4], the working alliance has also been considered a relevant factor to the specific context of coaching [5]. Indeed, findings from the recent meta-study of Grassmann et al. [6] support the important role of working alliance as a contributing factor to effectiveness in the particular context of coaching, although the included studies were correlational by design, prohibiting causal conclusions. Specifically, the quality of working alliance was shown to correlate positively with coaching outcomes overall (*r* = 0.41, *k* = 27.95%, CI [0.34, 0.48], *p* < 0.001) and strongest with affective outcomes (*r* = 0.53, 95%, CI [0.44, 0.60], *p* < 0.001).

The most widely used conceptualization of working alliance derives from Bordin’s [7] pan-theoretical model that moves beyond the original psychotherapeutic approaches of the construct [8]. This all-inclusive model of helping relationships views working alliance as an establishment between therapist and client that ensues from a holistic collaborative process, and that is fostered by (1) the quality of the bond between the therapist and client, (2) the consensus on the tasks to be realized, and (3) the mutual agreement on the goals (e.g., [7]). These dimensions are commonly assessed by rating scales such as the Working Alliance Inventory (WAI; 36 items; [9]), or the more parsimonious Working Alliance Inventory—Short form (WAI-S; 12 items; [10]). Research has established support for use of the WAI-S as a proxy measure for the WAI [11, 12]. Also, the WAI-S is a widely applied self-report questionnaire in studies concerning research in psychotherapy [1] and other helping professions. The adjustment of the wording of the WAI-S to suit coaching [13] consequently enabled measurement of working alliance for this specific context as well (e.g., [14–16]).

The dimensionality of the WAI-S has been delineated in a number of studies in contexts other than coaching and has yielded mixed results. As such, the originally proposed three dimensions [7] were supported in two studies involving parental training [17] and social services [12], as well as two studies in a counseling setting [18, 19]. Milot-Lapointe et al.’s [19] study also revealed a good fit for bilevel hierarchical models, that involved three first-order factors and a general alliance factor on the second level. Yet, other studies that were conducted in therapy contexts have reported either a two-factor structure, where the tasks and goals subscales were merged into a “contract” factor and the bond subscale formed the “contact” factor [20], or a unidimensional structure [21–23]. So far, only two studies described the factorial structure of the working alliance in a coaching context, more specifically sports coaching. These particular studies [24, 25] used an adjusted version of the WAI-S for sports coaching and found contrasting evidence for either a one- or three-factor structure. However, as the relationship between coaches and athletes represents an intense, asymmetrical power dynamic [26], the working alliance in sports coaching may deviate from the theoretical conceptualization by Bordin [7]. This hampers inferences from sports coaching contexts [i.e., 24, 25] regarding the factorial structure of the WAI-S to the broader context of coaching.

A possible explanation for contrasting findings regarding the WAI-S factor structure may be found in context-specific differences between interventions—i.e. therapy, counseling, coaching—that were studied [27], such as their target population [28], duration [2, 29], subject matter [2], orientation on either future or past experiences [30, 31], and degree to which personal backgrounds are explored [32]. Accordingly, coaching relationships have, for instance, been suggested to hold weaker emotional bonds [2] and maintain a lower emphasis on relational dynamics in comparison to therapeutic relationships [31].

Considering the prominent role of working alliance as a posited common factor in coaching’s effectiveness research [e.g., 33–35], it is essential to determine the psychometric validity of the WAI-S in terms of reflecting the theoretical dimensions of working alliance as specified by Bordin [7] in coaching. Either the scale adequately reflects the three dimensions and is accordingly considered an apt tool in its current form, or evidence of a divergent structure is found, which may warrant future alteration of the scale in addition to possible reconceptualization of the working alliance construct in coaching. Because of the scarcity of existing factorial research on this instrument in the specific context of coaching, the first goal of the current study is to investigate whether the three dimensions of working alliance (i.e., bond, tasks, goals) as proposed by Bordin [7] and measured by the WAI-S, represent the factorial structure of working alliance in a coaching setting.

### Working alliance dynamics and longitudinal measurement invariance

Additionally, temporal dynamics of working alliance across the coaching process are scarcely researched, as only few studies involving working alliance in coaching have included multiple assessments of the WAI-S over time (e.g., [36, 37]). Even though current studies involving working alliance in coaching are mainly cross-sectional by design, de Haan et al. [36] suggest that the quality of working alliance tends to increase as coaching sessions progress. In order to facilitate longitudinal investigations to better understand dynamics of working alliance in coaching, it is important to ascertain that observed respondent-reported scores on the WAI-S gauge the same underlying construct within the same sample across different points in time, also known as longitudinal measurement invariance (LMI; [38, 39]). In other words, to assess whether observed changes reflect actual change in working alliance rather than changes in its measurement. Therefore, testing for LMI is considered to be an essential procedure in psychometric validation [40, 41]. If the assumption of LMI does not hold, changes in the respondents’ assessment of the item(s) content may be confounded by a change in its perception over time, namely a response shift [39]. Such change may be due to “(a) a change in the respondent’s internal standards of measurement (scale recalibration); (b) a change in the respondent’s values (reprioritization); or (c) a redefinition of the target construct (reconceptualization)” [42, p. 1532]. In this case, any inferences about growth and change of latent constructs across measurements could be biased and inaccurate [40]. It is thus implied that LMI is a prerequisite for composing meaningful comparisons within the same sample across time [21, 38, 43]. LMI can be tested by analyzing the equality of the factor structure (i.e., factorial invariance) of a measure across time, which is preferable to simply taking for granted that the criteria for LMI are met [23, 41, 44, 45].

To date, only few longitudinal studies have investigated the WAI-S to assess possible changes in the working alliance over time. These studies were all situated in therapy [17, 21] and counseling contexts [19], and concluded that the WAI-S can be considered invariant over time within contexts alike. However, LMI of the WAI-S in the distinct context of coaching remains unexplored. Considering this, as well as the aforementioned existing inconsistencies with respect to the factorial structure of the WAI-S, further empirical investigation of these psychometric properties of the WAI-S in the particular context of coaching is warranted. Therefore, the second goal of this study is to investigate the LMI of the WAI-S in the context of coaching.

### The present study

Resuming, the aims of the present study are to, first, determine whether the three dimensions of working alliance (i.e., bond, tasks, goals) represent the factorial structure of working alliance, using the WAI-S. Second, this study aims to find out whether the scores obtained on the WAI-S are associated with measurement invariance as a function of time in a coaching context.

## Method

### Participants

The sample for this study consisted of Dutch speaking coaching clients. A total of 2,085 coachees completed the WAI-S at time one (T1) and 1,111 at time two (T2). Data screening resulted in a sample of *n* = 690 (T1) and *n* = 490 (T2; see also Results paragraph). The final sample (*N* = 490) was used to test longitudinal properties of the WAI-S between the two points of measurement. This sample comprised coachees between the ages of 18 and 64 years (*M* = 41.04, *SD* = 10.20), of whom 193 (39.4%) were male and 297 (60.6%) were female. A majority of these coachees completed higher vocational education (*n* = 197; 40.2%), were either married, in a registered partnership, or living together (*n* = 349; 71.2%), entered the coaching sessions on their own initiative (*n* = 264; 53.9%), and entered the coaching process on a voluntary basis (*n* = 456; 93.1%), contrary to being enrolled by, for example employers or benefit agencies (*n* = 28; 5.7%). Table 1 shows more detailed information about characteristics of the study sample.

**Table 1:** Sociodemographic and other characteristics of the sample (N = 490)

Characteristic	*n*	% Total
*Gender*		
Male	193	39.4
Female	297	60.6
*Educational level*		
PhD	9	1.8
University education	100	20.4
Higher vocational education	197	40.2
Pre-university education	7	1.4
Senior general secondary education	18	3.7
Secondary vocational education—specialized training	30	6.1
Secondary vocational education	93	19.0
Pre-vocational secondary education	14	2.9
Lower vocational education	9	1.8
Elementary school	1	.2
Other	7	1.4
Missing	5	1.0
*Marital status*		
Married	349	71.2
In a relationship, not living together	35	7.1
Single	100	20.4
Other	6	1.2
*Initiative for coaching*		
Medical officer	4	0.8
Myself	264	53.9
Manager	140	28.6
Human recourses (HR)	28	5.7
General practitioner (GP)	1	0.2
Other	53	10.8

### Study design and procedure

The study employed a two-wave (T1-T2) longitudinal survey design. Data were collected between March 2013 and April 2019 by the Dutch Association of Professional Coaches (Nederlandse Orde van Beroepscoaches; [NOBCO]). Independent coaches who were affiliated with NOBCO invited their coachees to digitally complete the WAI-S at two separate time points: after the intake (T1) and at an interim assessment halfway through the coaching procedure (T2). The time in between the two measurements varied depending on the number of agreed upon sessions, ranging from two to 92 sessions (*M* = 10.98, *SD* = 10.52, *Mdn* = 8) and encompassing a time period ranging from 21 to 750 days (i.e., zero to 24 months, *SD* = 119.32 days, *Mdn* = 178 days). Coaching was delivered through face-to-face (*n* = 310), online (i.e., via telephone, chat, email, text message, webcam or skype; *n* = 12), and blended modes (i.e., face-to-face mixed with online; *n* = 168), with an average duration of 84 min (*SD* = 23.19) per session. Applied coaching approaches mainly concerned development-oriented (*n* = 98; 20.0%), solution-oriented (*n* = 61; 12.4%), and cognitive coaching (*n* = 46; 9.4%; for a complete overview see Additional file 1: Table S1). Coaching processes had an overall focus on the enhancement of personal development and goal attainment, and improvement of well-being. All coachees provided digitally signed consent prior to completing the questionnaire and were informed about the goal, anonymity, confidentiality of the study, and possibility to withdraw at any time, without consequences.

### Measures

Working alliance was assessed at T1 and T2. The first assessment additionally covered sociodemographic and other characteristics of the sample, such as initiative for coaching and life satisfaction.

### Working Alliance Inventory—Short form, coachee version

The WAI-S [13] is a 12-item questionnaire that was created to measure satisfaction with the three domains of working alliance as proposed by Bordin [7], viewed from the coachee’s perspective. The present study used a Dutch translation of a version of the WAI-S (see Additional file 2) that was adapted for coaching by Baron and Morin [13]. The three subscales each correspond to, respectively, four items measuring the affective bond between coach and coachee (e.g., “My coach and I trust one another”); four items measuring the perceived agreement on tasks (e.g., “We agree on what is important for me to work on”); and four items measuring the perceived agreement on goals (e.g., “My coach and I are working towards mutually agreed upon goals”). Each item is rated on a 7-point Likert scale, ranging from never [= 1] to always [= 7]. The scores of two negatively worded items (WAI-S Items 9 and 11) were reversed, such that higher scores corresponded to higher satisfaction with working alliance. The WAI-S can also be used as a total scale that measures the respondents’ overall satisfaction with working alliance [13], with excellent internal consistency in the current sample (Cronbach’s *α*_*Time1*_ = 0.94, Cronbach’s *α*_*Time2*_ = 0.94).

### Control variables

As the number of sessions in between measurements differed between coachees, we included number of sessions, as registered by their coaches, as a control variable to the measurement invariance models. We additionally included life satisfaction at T1 as covariate, based on a meta-analytic review in counseling that suggests that it is a more strenuous task to develop a positive working alliance with clients who experience high levels of dissatisfaction with life [46]. We used a Dutch translation of the Satisfaction with Life Scale (SWLS; see Additional file 3) that was originally developed by Diener et al. [47], to assess the degree to which a coachee evaluates their own life as satisfying. The scale comprises five items (e.g., “The conditions of my life are excellent”) that are rated on a 7-point Likert scale ranging from strongly disagree [= 1] to strongly agree [= 7], with higher values indicating a higher level of satisfaction with life. With a Cronbach’s alpha value of 0.86, internal consistency of the scale was considered good in the current sample, which is in line with earlier findings on reliability for this translated scale (*α* = 0.82; [48]).

### Data analytic approach

Data were screened and descriptive statistical and dropout analysis were conducted using SPSS 28.0 [49]. Then, a series of confirmatory factor analyses (CFA) were performed in R 4.1.1 [50], using the Lavaan package (version 0.6–11; [51]). Considering the kurtosis distribution exceeded the cutoff value $\ge$ 2 [52], maximum likelihood estimation (robust to non-normality) was used by default. CFA examined model fit for a (1) one-factor model, (2) two-factor model (i.e., “contract-contact” factor, [20]), and (3) three-factor model, differentiating tasks, goals, and bond subscales. Model fit was evaluated by a set of parameters, including the *χ*^*2*^/*df* ratio, root mean square error of approximation (RMSEA; [53]), standardized root mean residual (SRMR; [54]); comparative fit index (CFI; [55]) and Tucker-Lewis index (TLI; [56]). A good model fit was considered to be reflected in a small (i.e., close to zero) *χ*^*2*^/*df* ratio [57], and CFI/TLI values greater than 0.90 [58]. RMSEA values < 0.06–0.08 are suggested to indicate good to acceptable fit, while values greater than 0.10 indicate poor fit [58–60]. SRMR values < 0.08 indicate good fit [58], although values < 0.10 are considered acceptable [61]. Standardized residual covariances were examined across the various models, while considering values less than ± 2.58 as indicative of good fit [62]. The chi-square difference test ($\Delta$ *χ2*) was ultimately able to determine a significantly better fit between competing models, resulting in a best fitting model that was used as a baseline model in the next step of testing LMI.

Next, multigroup confirmatory factor analysis (MGCFA) was used to test LMI, which in practice has become the standard for investigating measurement invariance in a structural equation model (SEM) framework [43]. LMI was investigated by sequentially testing a series of nested multigroup models on model fit with increasingly restrictive model constraints, using the lavaan package [51]. In doing so, we distinguished the three most commonly examined levels of measurement invariance [39]: (1) configural invariance, i.e., testing of the equality of pattern of fixed and free factor loadings, or equality of the factorial structure, across time; (2) metric invariance, i.e., testing of the equality of factor loadings of the items across time by setting the corresponding factor loadings to be equal across time, and (3) scalar invariance, i.e., testing of the equality of factor loadings and intercepts across time by constraining all intercepts to be equal across time. Since differences in fit indices are less affected by sample size than chi-square tests of measurement invariance [63], the present study compared nested models on meaningful differences in model fit by examining the changes in CFI ($\Delta$ CFI). We favored the recommendation by Meade et al. [63] to consider changes greater than 0.002 as an indicator for nonequivalence (i.e., a violation of measurement invariance) between nested models, over the more commonly used difference in CFI being greater than 0.01 (e.g., [64, 65]), because the latter threshold may be too tolerant for detecting certain forms of non-invariance [63]. When full invariance was not supported, we adhered to Vandenberg and Lance’s [39] advice to investigate partial invariance by exploring modification indices and selecting and individually freeing intercepts of items with large values to allow them to vary. This enabled us to determine which specific items were non-invariant and responsible for the differences in CFI. Following Cheung and Rensvold [66], non-invariant items were retained when at least partial measurement invariance could be established [67]. Lastly, raw and latent means for each subscale of the WAI-S were compared between T1 and T2, with differences expressed as Cohen’s d (i.e., standardized mean difference; [68]). All findings were interpreted against a significance threshold of *p* < 0.05.

### Post hoc sensitivity analysis

The main study findings demonstrated a best-fitting three-factorial structure of the WAI-S, and evidenced partial scalar invariance for this model, with and without inclusion of covariates (see Table 4). Therefore, in an attempt to further consolidate our findings by replication, we conducted a post hoc sensitivity analysis on the original samples that lacked additional information on covariates, and including both completers and dropouts, resulting in *n* = 1,986 at T1, and *n* = 1,020 at T2.

## Results

### Descriptive results

At T1, controlling for multivariate outliers through Mahalanobis distance resulted in the detection of 99 extreme cases (4.7%), which were excluded from the sample. Of the remaining 1986 participants, 1286 (64.8%) had missing data on the number of attended coaching sessions; in 9 cases (1.3%) the logged end date of the coaching trajectory preceded its start date; and in one case (0.1%) no coaching session was completed. After exclusion of these cases, the final sample at T1 consisted of *n* = 690 participants, of which *n* = 490 also provided complete data at T2.

Next, dropout-completer comparisons showed that coachees who dropped out from the study at T2 did not differ from completers with regard to gender, marital status, willingness to participate, educational level, and initiative to participate in the survey (respectively: χ^*2*^(1, *n* = 690) = 0.50, *p* = 0.480; χ^*2*^(3, *n* = 690) = 0.64, *p* = 0.888; χ^*2*^(1, *n* = 680) = 2.40, *p* = 0.121; Fisher’s Exact Test, *p* = 0.524; and *p* = 0.184). Furthermore, independent samples *t*-tests did not reveal any significant differences on the subscales of the WAI-S at T1 between dropouts and completers (Bond: *t*(688) = 0.23, *p* = 0.817; Tasks: *t*(688) = 1.03, *p* = 0.302; Goals: *t*(688) = 0.41, *p* = 0.684). Since these findings suggested random sample attrition at T2, participant data was omitted for coachees who did not complete the second survey (*n* = 200). This led to the final sample of *n* = 490 that was used to test longitudinal properties of the WAI-S between the two points of measurement.

At both time points, skewness distribution of the twelve WAI-S items was not problematic (Range: − 1.51 to 0.03 [T1]; − 0.98 to − 0.11 [T2]), however kurtosis contained exceeding cutoff values (> 2) for Item 11 (2.96 [T1]; 2.28 [T2]). The 12-item scale yielded strong correlations (Range: *r* = 0.65 to 0.82 [T1]; *r* = 0.68–0.81 [T2]) at the two time points for the Bond, Tasks and Goals factors, see Table 2.

**Table 2:** Factor correlations and reliability estimates for the 12-items WAI-S (N = 490)

Factor	1	2	3	4		6	7	8
1. Bond (T1)	—							
2. Bond (T2)	0.66*	—						
3. Tasks (T1)	0.82*	0.60*	—					
4. Tasks (T2)	0.53*	0.77*	0.61*	—				
5. Goals (T1)	0.65*	0.50*	0.79*	0.51*	—			
6. Goals (T2)	0.47*	0.68*	0.57*	0.81*	0.60*	—		
7. Number of sessions	0.00	− 0.03	0.01	− 0.03	− 0.03	− 0.06	—	
8. SWLS (T1)	0.22*	0.20*	0.20*	0.20*	0.21*	0.24*	− 0.12*	
Cronbach’s *α*	0.90	0.87	0.94	0.93	0.74	0.80	—	0.86

*SWLS * = satisfaction with life Scale; *T =* time. *p < 0.01

### Confirmatory factor analysis

Fit indices for all CFA models are presented in Table 3. The three-factor model was appointed as best fitting model at both T1 (χ^*2*^/*df* = 4.29, RMSEA = 0.089, CFI = 0.958, TLI = 0.945, SRMR = 0.043) and T2 (χ^*2*^/*df* = 4.46, RMSEA = 0.091, CFI = 0.952, TLI = 0.938, SRMR = 0.040), although RMSEA values could be considered suboptimal for all models tested. Standardized residual covariances for the three-factor model fell within the acceptable range, with the exception of a value of 4.97 for WAI-S Items 9 (“My coach does not understand what I am trying to accomplish in coaching”—goals factor) and 11 (“My coach and I have different ideas on what my problems are”—goals factor) at T1. Based on the CFA results, the three-factor model was considered as baseline model for measurement invariance testing.

**Table 3:** Fit indices for confirmatory factor analysis at T1 (N = 490) and T2 (N = 490)

CFA models	χ^*2a*^	*df*	χ^*2*^/*df*	RMSEA [90% CI]	CFI	TLI	SRMR	$\mathbf{\Delta}\mathbf{\upchi 2}$
*T1*								
One-factor	389*	54	7.21	0.123 [0.112; 0.135]	0.914**	0.895**	0.053**	
Two-factor	237*	53	4.47	0.092[0.080; 0.104]**	0.953**	0.942**	0.045**	107.07*
Three-factor	219*	51	4.29	0.089 [0.077; 0.102]**	0.958**	0.945**	0.043**	17.40*
*T2*								
One-factor	364*	54	6.74	0.118 [0.106; 0.129]	0.916**	0.898**	0.049**	
Two-factor	249*	53	4.70	0.094 [0.082; 0.106]**	0.947**	0.934**	0.041**	88.95*
Three-factor	227*	51	4.46	0.091 [0.079; 0.103]**	0.952**	0.938**	0.040**	23.90*

*df* = degrees of freedom; *RMSEA* = Robust root mean square error of approximation; *CI* = confidence interval; *CFI* = Robust comparative fit index; *TLI* = Robust Tucker-Lewis index; *SRMR* = standardized root mean residual

*p < .001; **Meeting threshold criteria: RMSEA/SRMR < .10, CFI/TLI > .90

^a^Rounded at nearest integer

### Longitudinal measurement invariance

All fit indices associated with measurement invariance solutions are presented in Table 4. The configural invariance model showed an adequate fit (χ^*2*^/*df* = 3.62, RMSEA = 0.079, CFI = 0.954, TLI = 0.939, SRMR = 0.039), and supported a similar factor structure across time. Then, the sequential test of metric invariance indicated no meaningful differences in model fit compared to the configural test (χ^*2*^/*df* = 3.55, RMSEA = 0.078, CFI = 0.952, TLI = 0.941, SRMR = 0.045, ΔCFI =  − 0.001), therefore metric invariance across time was assumed. Next, scalar invariance was tested and results showed that assumptions of scalar invariance were violated, χ^*2*^/*df* = 3.63, RMSEA = 0.079, CFI = 0.948, TLI = 0.940, SRMR = 0.047, ΔCFI =  − 0.004. When we tested for partial invariance by freeing the intercept of WAI-S Item 4 (“My coach and I trust one another”—bond factor), results revealed a good model fit (χ^*2*^/*df* = 3.49, RMSEA = 0.077, CFI = 0.951, TLI = 0.943, SRMR = 0.046), supporting partial scalar invariance (ΔCFI =  − 0.001). As can be read from Table 4, measurement invariance results were overall comparable with and without the covariates number of sessions and life satisfaction included in the measurement models.

**Table 4:** Fit indices for multigroup confirmatory factor analysis of the 12-item WAI-S, three-factor model, across time (N = 490)

Three-factor model	χ^*2d*^	*df*	χ^*2*^/*df*	RMSEA [90% CI]	CFI	TLI	SRMR	ΔCFI
*Model*^*a*^								
T1	219*	51	4.29	0.089 [0.077; 0.102]**	0.958**	0.945**	0.043**	
T2	277*	51	4.46	0.091 [0.079; 0.103]**	0.952**	0.938**	0.040**	
Configural	446*	102	4.38	0.090 [0.082; 0.099]**	0.955**	0.942**	0.041**	
Metric	470*	111	4.23	0.088 [0.080; 0.096]**	0.954**	0.945**	0.049**	− 0.001
Scalar	515*	120	4.29	0.088 [0.080; 0.096]**	0.950**	0.945**	0.051**	− 0.004
Partial scalar	486*	118	4.12	0.086 [0.078; 0.094]**	0.953**	0.947**	0.050**	− 0.001
*Model*^*b*^								
T1	229*	60	3.81	0.082 [0.071; 0.094]**	0.958**	0.945**	0.040**	
T2	234*	60	3.90	0.084 [0.073; 0.095]**	0.952**	0.938**	0.038**	
Configural	462*	120	3.85	0.083 [0.075; 0.091]**	0.955**	0.942**	0.039**	
Metric	485*	129	3.76	0.081 [0.074; 0.089]**	0.954**	0.944**	0.046**	− 0.001
Scalar	530*	138	3.84	0.082 [0.075; 0.090]**	0.950**	0.943**	0.049**	− 0.004
Partial scalar	501*	136	3.69	0.080 [0.072; 0.087]**	0.953**	0.946**	0.048**	− 0.001
*Model*^*c*^								
T1	249*	69	3.61	0.079 [0.068; 0.090]**	0.956**	0.942**	0.040**	
T2	250*	69	3.63	0.080 [0.069; 0.090]**	0.951**	0.936**	0.038**	
Configural	499*	138	3.62	0.079 [0.072; 0.087]**	0.954**	0.939**	0.039**	− 0.001
Metric	522*	147	3.55	0.078 [0.071; 0.085]**	0.952**	0.941**	0.045**	− 0.004
Scalar	566*	156	3.63	0.079 [0.072; 0.086]**	0.948**	0.940**	0.047**	− 0.001
Partial scalar	538*	154	3.49	0.077 [0.070; 0.084]**	0.951**	0.943**	0.046**	

^*a*^No covariates; ^*b*^Covariate = number of sessions; ^*c*^Covariates: number of sessions and life satisfaction; ^*d*^Rounded at nearest integer; *df* = degrees of freedom; RMSEA = Robust root mean square error of approximation; *CI* = confidence interval; *CFI* = Robust comparative fit index; *TLI* = Robust Tucker-Lewis index; *SRMR* = standardized root mean residual

*p < 0.001; ^**^Meeting threshold criteria: RMSEA/SRMR $<$ .10, CFI/TLI $>$ .90

### Raw and latent mean comparisons

Raw means for the bond, tasks and goals subscales were significantly higher at T2 compared to T1 (Bond: *t*(489) = 11.32, *p* < 0.001, *d* = 0.51; Tasks: *t*(489) = 9.36, *p* < 0.001, *d* = 0.42; Goals: *t*(489) = 8.65, *p* < 0.001, *d* = 0.39; see also Table 5). Standardized latent factor intercepts, extracted from the partial scalar measurement model without covariates, also suggested an increase from T1 to T2 for bond (*d* = 0.24, *p* < 0.001), tasks (*d* = 0.27, *p* < 0.001), and goals (*d* = 0.26, *p* < 0.001), as was observed for the model that included ‘number of sessions’ as covariate (Bond: *d* = 0.18, *p* < 0.001; Tasks: *d* = 0.20, *p* < 0.001; Goals: *d* = 0.17, *p* < 0.001), although less pronounced. Additional inclusion of ‘life satisfaction’ as covariate to the partial scalar invariance model resulted in a considerable decline in effect size estimates, suggesting only a significant increase from T1 to T2 for the bond factor (*d* = 0.09, *p* = 0.044), but not for tasks (*d* = 0.09, *p* = 0.057) or goals (*d* = 0.05, *p* = 0.241).

**Table 5:** Overview of the standardized factor loadings and descriptive statistics for the baseline measurement model of the 12-item WAI-S (N = 490)

	T1			T2			
WAI-S Item	load	*M*	*SD*	load	*M*	*SD*	*d*
*Bond*		5.36	0.99		5.75	.85	0.51
1. I believe my coach likes me	0.78			0.75			
2. I am confident in my coaches’ ability to help me	0.85			0.80			
3. I feel my coach appreciates me	0.87			0.80			
4. My coach and I trust one another	0.83			0.81			
*Tasks*		5.48	0.97		5.82	0.83	0.42
5. My coach and I agree about the things I will need to do in coaching to help improve my situation	0.90			0.86			
6. What I am doing in coaching gives me new ways of looking at my problem	0.88			0.88			
7. We agree on what is important for me to work on	0.90			0.90			
8. I believe the way we are working							
with my problem is correct	0.87			0.86			
*Goals*		5.64	0.83		5.91	0.72	0.39
9. My coach does not understand what I am trying to accomplish in coaching (r)	0.47			0.59			
10. My coach and I are working towards mutually agreed upon goals	0.83			0.78			
11. My coach and I have different ideas on what my problems are (r)	0.39			0.55			
12. We have established a good understanding of changes that would be good for me	0.83			0.87			

All factor loadings *p* < 0.001; *M*, *SD* = raw mean and standard deviation; *r* = reverse coded item; *d =* standardized mean difference (T2-T1)

### Post hoc sensitivity analysis

Results from post hoc sensitivity analysis (see Additional file 4: Tables S1–S5) approximated our main study findings, corroborating that results were not systematically influenced by study attrition. The three-factor model of the WAI-S best fitted the data at both measurements (T1: χ^*2*^/*df* = 12.77, RMSEA = 0.084, CFI = 0.960, TLI = 0.948, SRMR = 0.037; T2: χ^*2*^/*df* = 8.66, RMSEA = 0.093, CFI = 0.950, TLI = 0.935, SRMR = 0.039). Moreover, partial scalar invariance for the WAI-S was demonstrated (χ^*2*^/*df* = 10.17, RMSEA = 0.084, CFI = 0.954, TLI = 0.948, SRMR = 0.044, ΔCFI =  − 0.001), identifying two items (WAI-S Items 2 [“I am confident in my coaches’ ability to help me”—bond factor] and 6 [“What I am doing in coaching gives me new ways of looking at my problem”—tasks factor]) as non-invariant (conform Meade et al.’s [63] threshold of measurement invariance: $\Delta$ CFI > 0.002). Raw means were higher at T2 compared to T1 for bond (*t*(2363) =  − 13.13, *p* < 0.001, *d* = 0.53), tasks (*t*(2363) =  − 10.85, *p* < 0.001, *d* = 0.43), and goals (*t*(2325) =  − 12.46, *p* < 0.001, *d* = 0.46). Standardized latent factor intercepts, extracted from the partial scalar measurement model, also suggested an increase from T1 to T2 for bond (*d* = 0.38, *p* < 0.001), tasks (*d* = 0.34, *p* < 0.001), and goals (*d* = 0.33, *p* < 0.001).

## Discussion

The first objective of this study was to determine whether the three dimensions of working alliance (i.e., Bond, Tasks and Goals) represent the factorial structure of working alliance in a coaching setting, using the Dutch translation of the measure WAI-S. By applying a standard CFA-framework that sequentially employed the testing of one-, two-, and three-factor structures of working alliance throughout sessions, our main, as well as post hoc findings, indicated that the three-factor model of the WAI-S most adequately represented our data. These results are in line with most research on this topic which delineated a multidimensional structure of the WAI-S (i.e., three factors; [12, 17–19, 25]), which is congruent with Bordin’s [7] original conceptualization of working alliance. Similar to Hukkelberg and Ogden’s [17] study results, we found Item 9 (“My coach does not understand what I am trying to accomplish in coaching”—goals factor) and Item 11 (“My coach and I have different ideas on what my problems are"—goals factor) to have relatively weak factor loadings [58, 69]. Moreover, we found these items to exhibit relatively high residual covariance, which may be due to overlap in item content, their proximity (i.e., serial order), as well as their negatively-keyed phrasing [70]. Indeed, recent research has evidenced a method effect related to the negatively worded items on the WAI-S in a therapeutic context [21], and researchers should be aware of specific psychometric challenges posed by these items, as previously pointed out by Mallinckrodt and Tekie [71].

Additionally, the present study revealed high intercorrelations between factors (i.e., ranging from 0.65 to 0.82), which render meaningful factor differentiation questionable. In order to gauge the degree to which multidimensionality influences the interpretation of (sub)scale scores, future researchers may consider to apply bifactor modelling by separating the general construct from domain specific factors [72, 73]. To our knowledge, Milot-Lapointe et al.’s [19] study that was conducted in a context of counseling, is unique in demonstrating a three-factor as well as bilevel representation of the WAI-S. Other studies [21, 24] attempting to specify a bifactor model for the WAI-S, failed due to problems with model convergence and identification, which may reflect model overspecification. Future researchers who encounter such issues are advised to evaluate parsimonious models with e.g., fewer group factors or clustered models that include correlated factors [74].

The second objective of this study was to investigate the longitudinal measurement invariance of the WAI-S. Meade et al.’s [63] suggested threshold of 0.002 allowed us to expand our range to detect nonequivalence. Using this threshold, we found evidence through MGCFA for the items of the WAI-S to be invariant across time at the levels of factor structure and loadings. When intercepts were constrained, a single non-invariant item that contributed to a worse model fit was identified: Item 4 (“My coach and I trust one another”—bond factor). This led us to surmise partial scalar invariance, namely a change in pattern of item intercept. Additional post hoc analysis marked Item 2 (“I am confident in my coaches’ ability to help me”—bond factor) as well as Item 6 (“What I am doing in coaching gives me new ways of looking at my problem”—tasks factor) as non-invariant. It is proposed that the presence of a single invariant scale-item [67], or a scale that is evenly split in invariant and non-invariant items [73], nevertheless allows for meaningful interpretations between groups. Moreover, it should be noted that when we would have adhered to the more commonly used threshold of $\Delta$ CFI $\ge$ 0.01 instead of 0.002, a lack of invariance would not have been detected. On these notions, we consider the WAI-S to be useful in capturing the working alliance construct across coaching sessions, which permits us to make relevant comparisons of the WAI-S scores across different measurement moments. Hence, based on our current findings it can be assumed that a temporal fluctuation in observed scores on the WAI-S represents an actual change in the level of working alliance as experienced by coachees, in line with previous conclusions of researchers in different contexts [17, 19, 21].

Furthermore, our findings suggest that coachees tend to score higher on all three subscales of the WAI-S as time progresses, in line with previous work suggesting that levels of working alliance are likely to grow with the development of the coaching process [36]. However, this does not necessarily prelude a linear course in the progression of working alliance throughout the remainder of the coaching process. Not only does literature suggest that a working alliance takes time to establish [75], working alliance has also been known to evolve, as well as to deteriorate along the road of treatment [76].

## Implications

The current findings have several practical implications for coaching researchers and practitioners who use WAI-S scorings to chart working alliance development. Given the importance of the construct of working alliance to the realization of coaching outcome [6], it is essential for research on working alliance to use a measure that promises valid inferences between sessions. Our findings assert that assessment of quality of a three-dimensional working alliance throughout coaching by means of interpreting and comparing the observed scores on the WAI-S appears to be a justifiable practice, albeit with some considerations in mind. This may incite researchers to make more resolute and determined interpretations regarding the accuracy of measurements on the WAI-S. In all, we believe this study may serve as a good starting point for future longitudinal research on working alliance across the process of coaching, to further the overall grasp on the relationship between temporal dynamics of working alliance and coaching effectiveness.

Given this study’s findings, we now may understand the WAI-S for coaching to comprise a three-factorial structure, as proposed by Bordin [7], allowing coaches to tap into these dimensions in order to realize the coachees’ potential of experienced working alliance. As such, it can be questioned what actions coaches can concretely undertake themselves in between sessions to put these gained insights to good use. In other words: how can a coach maintain (or even increase) a qualitatively good working alliance on a total and subscale level? Although this question is beyond the scope of this study, we do point out that test users should be conscious about the fact that instruments are complementary to the practitioner’s expertise, instead of being independently used for decision making [77]. Hence, we deem it of importance for coaches to not only discuss a possible change in test scores of working alliance dimensions across sessions with their coachees, but to also qualitatively enrich the interpretation of such detected changes by discussing what the coachee experiences as fair developments and room for further advancement. Then, more substantial insights can be gained on for instance, possible causes for a difficult development of working alliance in the early stages of coaching, or on specific changes in working alliance during coaching that were experienced as more positive or negative. A deepened understanding of working alliance development in coaching may help to more concretely identify and/or develop specific tools or techniques to optimize it directly, or indirectly through related factors, and WAI-S scores may serve as a source of information.

For now, coaches may notify their coachees for potential developments on facets of working alliance along the coaching process by informing them at the start of coaching on such possible occurrences, and create or sustain an environment where experiences on working alliance developments can be voiced and discussed freely.

## Limitations and recommendations for future research

This study has a number of distinctive strengths, such as being one of the first to investigate the factorial structure of WAI-S in coaching; first to examine longitudinal measurement invariance of the WAI-S in a broad coaching context; having a large sample size. Herewith, there are several limitations that should be marked when interpreting our findings.

First, the sample was collected by a professional body of coaches, which limits the findings to a specific group of coachees. Also, coachees were allowed to decide for themselves whether or not they participated in the survey, possibly introducing self-selection bias. Because non-probability sampling often involves a biased sample with findings that cannot be generalized to the general population [78], future studies should replicate our findings by recruiting a random sample of coachees that may be more representative of the general population of coachees.

Second, since our study design was limited to two measurements, we are uncertain if our findings on LMI of the WAI-S would hold when measured at additional time points. Therefore, we encourage future researchers on the temporal dynamics of working alliance in coaching to include additional points of measurements in longitudinal study designs and try to replicate our study findings.

Third, to date no extensive validation research has been performed on the Dutch translation and adaptation of the WAI-S to the coaching context. Following Brislin’s [79] classic model for translation and validation of cross-cultural research, we suggest future researchers to apply the method of back-translation, which allows for the identification of translation errors. Moreover, the measure would benefit from additional research on construct validity, which may ensure researchers and coaches working with the WAI-S that the items in the scale capture all relevant aspects of working alliance in coaching.

Fourth and finally, we adhered to a relatively liberal RMSEA threshold (< 0.10) in comparison to more stringent cutoffs used in previous studies [i.e., 19, 20, 22]. Although we used a combination of multiple fit indices and theoretical considerations to interpret CFA results, we recognize that this approach allowed us to specify a model (i.e., three-factor model) with acceptable, opposed to perfect, fit to the data. Additionally, although freeing correlated residuals (post hoc) would have improved model fit considerably, we did not follow this approach because it hampers replication across independent samples [80].

## Conclusion

The main purpose of the present study was to determine whether the scores obtained on the WAI-S were associated with measurement invariance as a function of time in a coaching context. Also, we aimed to determine whether the three dimensions of working alliance (i.e., bond, tasks, goals) represent the factorial structure of working alliance, using the WAI-S. This study provided evidence of a three-factor structure (Bond, Tasks, Goals; Bordin [7]) of the WAI-S in a coaching context. Moreover, due to the detection of one non-invariant item, we found evidence of partial measurement invariance across coaching sessions. This rendered our finding of increased WAI-S scores on the three dimensions in this sample to be meaningful, suggesting a positive development of the experienced quality of the working alliance as coaching progressed. Practitioners and researchers may thus employ the WAI-S to quantitatively gauge the level of working alliance across time, but are advised to complement this with additional (qualitative) investigations, as well as a theoretically informed decision-making process regarding model specification, in order to validate and enrich interpretation. Since few researchers have preceded us in engaging into research concerning these psychometric features of the WAI-S in coaching, we believe our research makes a valuable contribution to evidence-based coaching practice by demonstrating that the WAI-S for coaching (although still in need for further investigation) can be used to accurately assess working alliance across coaching sessions.

## Supplementary Information

**Additional file 1.** Overview of the main types of coaching included in this study.

**Additional file 2.** Dutch translation of the WAI-S.

**Additional file 3.** Dutch translation of the SWLS.

**Additional file 4.** Results from post hoc sensitivity analysis.

## Data Availability

The data that support the findings of this study are available from NOBCO but are not publicly available due to intellectual property restrictions. Data are however available from the corresponding author MS, upon reasonable request and with permission of NOBCO.

## Abbreviations

CI: Confidence interval
CFA: Confirmatory factor analysis
CFI: Comparative fit index
LMI: Longitudinal measurement invariance
MGCFA: Multi-group confirmatory factor analysis
RMSEA: Root mean squared error of approximation
SEM: Structural equation modeling
SRMR: Standardized root mean squared residual
SWLS: Satisfaction with life scale
TLI: Tucker Lewis index
WAI: Working alliance inventory
WAI-S: Working alliance inventory—short
