# Supplementary material for: Longitudinal measurement invariance of the Working Alliance Inventory - Short form across coaching sessions
Source: BMC Psychol. 2022 Nov 23;10:277. doi: 10.1186/s40359-022-00968-5 (PMC9685860; doi:10.1186/s40359-022-00968-5)
Supplement: Supplementary file 1 — Additional file 1. Overview of the main types of coaching included in this study. [file 40359_2022_968_MOESM1_ESM.docx]

Additional file 1

Overview of the main types of coaching included in this study (Supplementary Table 1).

| **Supplementary Table 1.** Main types of coaching delivered to the sample at T1 (N = 490) | | |
| --- | --- | --- |
|  | *n* | % Total |
| Development-oriented coaching | 98 | 20.0 |
| Solution-oriented coaching | 61 | 12.4 |
| Cognitive coaching | 46 | 9.4 |
| Cognitive-behavioristic coaching | 33 | 6.7 |
| Narrative coaching | 27 | 5.5 |
| Co-active coaching | 19 | 3.9 |
| Behavioristic coaching | 18 | 3.7 |
| Positive psychology coaching | 14 | 2.9 |
| Goal-focused coaching | 13 | 2.7 |
| Integral coaching | 13 | 2.7 |
| Other^a^ | 147 | 30.0 |
| Missing | 1 | .2 |
| ^a^Aggregated scores of cultural, energetic, existential, gestalt, humanistic, mentalizing, narrative, provocative, transpersonal, The Work, voice dialogue, walking and nature, psychodynamic, Socratic, systemic, person-centered, and entity-oriented coaching. | | |
