# Supplementary material for: Longitudinal measurement invariance of the Working Alliance Inventory - Short form across coaching sessions
Source: BMC Psychol. 2022 Nov 23;10:277. doi: 10.1186/s40359-022-00968-5 (PMC9685860; doi:10.1186/s40359-022-00968-5)
Supplement: Supplementary file 2 — Additional file 2. Dutch translation of the WAI-S. [file 40359_2022_968_MOESM2_ESM.docx]

Additional file 2

Working Alliance Inventory Short form – Coaching (WAI-S; Baron & Morin, 2009)

Dutch translation of the WAI-S (Waringa, 2013) by: Waringa, Ribbers, and Naaborg (2013)

Introductie
Hieronder volgt een reeks uitspraken die uiteenlopende gevoelens of gedachten omschrijven die u kunt hebben met betrekking tot uw coach. Geeft u alstublieft aan in hoeverre elk van deze uitspraken weergeeft hoe u er zelf over denkt of wat u zelf voelt.

1. Ik denk dat mijn coach me aardig vindt.

| 1 | 2 | 3 | 4 | 5 | 6 | 7 |
| --- | --- | --- | --- | --- | --- | --- |
| Nooit | Zelden | Soms | Geregeld | Vaak | Zeer vaak | Altijd |

2. Ik heb er vertrouwen in dat mijn coach in staat is om mij te helpen.

| 1 | 2 | 3 | 4 | 5 | 6 | 7 |
| --- | --- | --- | --- | --- | --- | --- |
| Nooit | Zelden | Soms | Geregeld | Vaak | Zeer vaak | Altijd |

3. Ik heb het gevoel dat mijn coach me waardeert.

| 1 | 2 | 3 | 4 | 5 | 6 | 7 |
| --- | --- | --- | --- | --- | --- | --- |
| Nooit | Zelden | Soms | Geregeld | Vaak | Zeer vaak | Altijd |

4. Mijn coach en ik vertrouwen elkaar.

| 1 | 2 | 3 | 4 | 5 | 6 | 7 |
| --- | --- | --- | --- | --- | --- | --- |
| Nooit | Zelden | Soms | Geregeld | Vaak | Zeer vaak | Altijd |

5. Mijn coach en ik zijn het eens over de stappen die gezet moeten worden om mijn situatie te verbeteren.

| 1 | 2 | 3 | 4 | 5 | 6 | 7 |
| --- | --- | --- | --- | --- | --- | --- |
| Nooit | Zelden | Soms | Geregeld | Vaak | Zeer vaak | Altijd |

6. Mijn coach en ik hebben allebei vertrouwen in de zinvolheid van waar wij momenteel mee bezig zijn.

| 1 | 2 | 3 | 4 | 5 | 6 | 7 |
| --- | --- | --- | --- | --- | --- | --- |
| Nooit | Zelden | Soms | Geregeld | Vaak | Zeer vaak | Altijd |

7. We zijn het eens over wat belangrijk is voor mij om aan te werken.

| 1 | 2 | 3 | 4 | 5 | 6 | 7 |
| --- | --- | --- | --- | --- | --- | --- |
| Nooit | Zelden | Soms | Geregeld | Vaak | Zeer vaak | Altijd |

8. Ik denk dat onze werkwijze de juiste is om mijn ontwikkeling te ondersteunen.

| 1 | 2 | 3 | 4 | 5 | 6 | 7 |
| --- | --- | --- | --- | --- | --- | --- |
| Nooit | Zelden | Soms | Geregeld | Vaak | Zeer vaak | Altijd |

9. Ik heb twijfels over wat we proberen te bereiken.

| 1 | 2 | 3 | 4 | 5 | 6 | 7 |
| --- | --- | --- | --- | --- | --- | --- |
| Nooit | Zelden | Soms | Geregeld | Vaak | Zeer vaak | Altijd |

10. Mijn coach en ik werken toe naar doelen die we samen overeengekomen zijn.

| 1 | 2 | 3 | 4 | 5 | 6 | 7 |
| --- | --- | --- | --- | --- | --- | --- |
| Nooit | Zelden | Soms | Geregeld | Vaak | Zeer vaak | Altijd |

11. Mijn coach en ik hebben verschillende ideeën over wat ik nodig heb voor mijn ontwikkeling.

| 1 | 2 | 3 | 4 | 5 | 6 | 7 |
| --- | --- | --- | --- | --- | --- | --- |
| Nooit | Zelden | Soms | Geregeld | Vaak | Zeer vaak | Altijd |

12. We hebben samen een goed inzicht ontwikkeld in het soort veranderingen dat goed voor mij zou zijn.

| 1 | 2 | 3 | 4 | 5 | 6 | 7 |
| --- | --- | --- | --- | --- | --- | --- |
| Nooit | Zelden | Soms | Geregeld | Vaak | Zeer vaak | Altijd |
