# Supplementary material for: Longitudinal measurement invariance of the Working Alliance Inventory - Short form across coaching sessions
Source: BMC Psychol. 2022 Nov 23;10:277. doi: 10.1186/s40359-022-00968-5 (PMC9685860; doi:10.1186/s40359-022-00968-5)
Supplement: Supplementary file 3 — Additional file 3. Dutch translation of the SWLS. [file 40359_2022_968_MOESM3_ESM.docx]

Additional file 3

Satisfaction with Life Scale (SWLS; Diener et al., 1985)

Dutch translation of the SWLS by: Arrindell, Meeuwesen en Huyse (1991)

Introductie
Hieronder staat een aantal uitspraken. Geef aan in hoeverre onderstaande uitspraken op u van toepassing zijn. U kunt kiezen uit zeven antwoordmogelijkheden. Geef per vraag aan in welke mate u het eens of oneens

De zeven antwoordmogelijkheden zijn: 1: zeer mee oneens 2: mee oneens 3: enigszins mee oneens 4: neutraal 5: enigszins mee eens 6: mee eens 7: zeer mee eens.

1. Mijn leven is ideaal in de meeste opzichten.
2. Mijn levensomstandigheden zijn ideaal.
3. Ik ben tevreden met mijn leven, alles bij elkaar genomen.
4. De belangrijkste dingen die ik van het leven verwacht, heb ik tot nu toe ook gekregen.
5. Als ik mijn leven opnieuw zou leven, zou ik heel weinig anders doen.
