# Supplementary material for: Longitudinal measurement invariance of the Working Alliance Inventory - Short form across coaching sessions
Source: BMC Psychol. 2022 Nov 23;10:277. doi: 10.1186/s40359-022-00968-5 (PMC9685860; doi:10.1186/s40359-022-00968-5)
Supplement: Supplementary file 4 — Additional file 4. Results from post hoc sensitivity analysis. [file 40359_2022_968_MOESM4_ESM.docx]

Additional file 4

Results from post hoc sensitivity analysis (Supplementary Tables 2 – 5).

**Supplementary Table 2.** Sociodemographic and other characteristics of the sample at T1 (N = 1,986) and T2 (N = 1,020)

|  | T1 | | T2 | |  |
| --- | --- | --- | --- | --- | --- |
| Characteristic | *n* | % Total | *n* | % Total | χ*^2^* |
| Gender |  |  |  |  | .16, n.s. |
| Male | 794 | 40.0 | 400 | 39.2 |  |
| Female | 1192 | 60.0 | 620 | 60.8 |  |
| Educational level |  |  |  |  | 4.17, n.s. |
| PhD | 25 | 1.3 | 16 | 1.6 |  |
| University education | 477 | 24.0 | 246 | 24.1 |  |
| Higher vocational education | 817 | 41.1 | 433 | 42.5 |  |
| Pre-university education | 26 | 1.3 | 12 | 1.2 |  |
| Senior general secondary education | 74 | 3.7 | 32 | 3.1 |  |
| Secondary vocational education – specialized  Training | 102 | 5.1 | 49 | 4.8 |  |
| Secondary vocational education | 326 | 16.4 | 174 | 17.1 |  |
| Pre-vocational secondary education | 52 | 2.6 | 23 | 2.3 |  |
| Lower vocational education | 26 | 1.3 | 14 | 1.4 |  |
| Elementary school | 2 | .1 | 1 | .1 |  |
| Other | 32 | 1.6 | 11 | 1.2 |  |
| Missing | 27 | 1.4 | 8 | .8 |  |
| Marital status |  |  |  |  | .99, n.s.. |
| Married | 1397 | 70.3 | 722 | 70.8 |  |
| In a relationship, not living together | 162 | 8.2 | 79 | 7.7 |  |
| Single | 400 | 20.1 | 209 | 20.5 |  |
| Other | 27 | 1.4 | 10 | 1.0 |  |
| Initiative for coaching |  |  |  |  | 4.36, n.s. |
| Medical officer | 15 | .8 | 8 | .8 |  |
| Myself | 1217 | 61.3 | 606 | 59.4 |  |
| Manager | 430 | 21.7 | 249 | 24.4 |  |
| Human Recourses (HR) | 107 | 5.4 | 59 | 5.8 |  |
| General Practitioner (GP) | 3 | .2 | 2 | .2 |  |
| Other | 214 | 10.08 | 96 | 9.2 |  |

Note. n.s. = not significant (*p* ≥ .05).

**Supplementary Table 3.** Factor correlations and reliability estimates

for the 12-items WAI-S at T1 (N = 1,986) and T2 (N = 1,020)

| Factor | 1 | 2 | 3 | 4 | 5 | 6 |
| --- | --- | --- | --- | --- | --- | --- |
| 1. Bond (T1) | — |  |  |  |  |  |
| 2. Bond (T2) | -.03 | — |  |  |  |  |
| 3. Tasks (T1) | .79* | .01 | — |  |  |  |
| 4. Tasks (T2) | -.00 | .78* | .02 | — |  |  |
| 5. Goals (T1) | .67* | .03 | .80* | .04 | — |  |
| 6. Goals (T2) | .04 | .68* | .04 | .81* | .08* | — |
| Cronbach’s *α* | .88 | .87 | .93 | .92 | .75 | .79 |

Note. **p* < .01.

| **Supplementary Table 4.** Fit indices for confirmatory factor analysis at T1 (N = 1,986) and T2 = (N = 1,020) | | | | | | | | |
| --- | --- | --- | --- | --- | --- | --- | --- | --- |
| CFA models | χ*^2a^* | *df* | χ*^2^*/*df* | RMSEA  [90% CI] | CFI | TLI | SRMR | $\Delta\chi2$ |
| T1 |  |  |  |  |  |  |  |  |
| One-factor | 1201* | 54 | 22.24 | .114 [.109; .120] | .922** | .904** | .047** |  |
| Two-factor | 695* | 53 | 13.12 | .086[.080; .092]** | .957** | .946** | .039** | 333.14* |
| Three-factor | 651* | 51 | 12.77 | .084 [.079; .090]** | .960** | .948** | .037** | 41.73* |
| T2 |  |  |  |  |  |  |  |  |
| One-factor | 705* | 54 | 13.05 | .117 [.109; .125] | .916** | .897** | .047** |  |
| Two-factor | 481* | 53 | 9.08 | .096 [.088; .103]** | .945** | .931** | .041** | 179.38* |
| Three-factor | 442* | 51 | 8.66 | .093 [.085; .101]** | .950** | .935** | .039** | 39.41* |

Note. *^a^*Rounded at nearest integer; df = degrees of freedom; RMSEA = Robust root mean square error of approximation; CI = confidence interval; CFI = Robust comparative fit index; TLI = Robust Tucker-Lewis index; SRMR = standardized root mean residual; **p* < .001; ^**^Meeting threshold criteria: RMSEA/SRMR $<$ .10, CFI/TLI $>$ .90.

**Supplementary Table 5.** Fit indices for multigroup confirmatory factor analysis of the 12-item WAI-S, three-factor model, across time

| Three-factor model | χ*^2a^* | *df* | χ*^2^*/*df* | RMSEA  [90% CI] | CFI | TLI | SRMR | ΔCFI |
| --- | --- | --- | --- | --- | --- | --- | --- | --- |
| Model  T1  (*N* = 1,986)  T2  (*N* = 1,020)  Configural  Metric  Scalar  Partial scalar | 651*  442*  1097*  1144*  1256*  1201* | 51  51  102  111  120  118 | 12.77  8.66  10.75  10.31  10.47  10.17 | .084 [.079; .090]**  .093 [.085; .101]**  .087 [.083; .092]**  .085 [.080; .089]**  .085 [.081; .089]**  .084 [.080; .088]** | .960**  .950**  .956**  .955**  .951**  .954** | .948**  .935**  .944**  .947**  .946**  .948** | .037**  .039**  .038**  .043**  .046**  .044** | -.001  -.004  -.001 |

Note. *^a^*Rounded at nearest integer; df = degrees of freedom; RMSEA = Robust root mean square error of approximation; CI = confidence interval; CFI = Robust comparative fit index; TLI = Robust Tucker-Lewis index; SRMR = standardized root mean residual; **p* < .001; ^**^Meeting threshold criteria: RMSEA/SRMR $<$ .10, CFI/TLI $>$ .90.
